# Pan-cancer analysis reveals TRA16 as a master regulator of human carcinogenesis

**DOI:** 10.3389/fonc.2025.1543419

**Published:** 2025-05-13

**Authors:** Yanyan Zhu, Yike Gao, Xiaoqing Huang, Bowang Chen, Xinyi Wang, Ying Wu, Jian Sun, Xiaoyun Huang

**Affiliations:** ^1^ Department of Oncology, Henan Provincial People’s Hospital, People’s Hospital of Zhengzhou University, School of Clinical Medicine, Henan University, Zhengzhou, China; ^2^ Department of Pathology, Peking Union Medical College Hospital, Chinese Academy of Medical Science and Peking Union Medical College, Beijing, China; ^3^ Intelliphecy Center for Systems Medicine, Intelliphecy, Shenzhen, China; ^4^ Department of Data Science, Intelliphecy, Nanjing, China; ^5^ Department of Dermatology, Southern University of Science and Technology Yantian Hospital, Shenzhen, China; ^6^ Department of Obstetrics and Gynecology, Southern University of Science and Technology Yantian Hospital, Shenzhen, China; ^7^ College of Life Sciences, Hunan Normal University, Changsha, China

**Keywords:** oncogene, nuclear receptor, single cell, systems medicine, pan-cancer analysis

## Abstract

**Introduction:**

The nuclear receptor TR4 binding protein, TRA16, has been implicated in lung carcinogenesis; however, its broader role across diverse human cancers remains poorly understood. Understanding TRA16’s involvement in cancer biology could uncover novel regulatory mechanisms and potential therapeutic targets.

**Methods:**

We conducted a comprehensive pan-cancer analysis of TRA16 expression and function across multiple human malignancies. Gene co-expression networks, pathway enrichment, transcription factor analysis, organoid modeling, and intercellular communication profiling were employed. Tumor mutation burden (TMB) and microenvironmental features were also assessed in relation to TRA16 expression, stratified by TP53 mutation status.

**Results:**

Correlation analysis identified the cell cycle as the top enriched pathway among TRA16-associated genes, with key transcription factors, including RB-E2F, MYC, and TP53, regulating genes co-expressed with TRA16. In liver cancer organoid models, TRA16 and its co-expressed genes were significantly upregulated. Intercellular communication analysis showed that TRA16-positive cells exhibited increased autocrine signaling and overall signaling activity. Importantly, patients with high TRA16 expression demonstrated elevated TMB and decreased stromal and immune features.

**Discussion:**

These findings highlight TRA16 as a potential master regulator of oncogenic processes, contributing to tumor progression through coordinated regulation of cell cycle genes, intercellular signaling, and genomic instability. Our results provide new insights into TRA16’s role across cancers and support its potential as a novel oncogene.

## Introduction

Nuclear receptors are a family of transcription factors characterized by their ligand-binding and DNA-binding domains. Ligand binding to nuclear receptors enables the transactivation of downstream genes that play key roles in shaping and maintaining the hallmarks of cancer (1). For instance, a significant proportion of breast cancers express estrogen and progesterone receptors, both of which are crucial for cancer cell proliferation (2). Similarly, prostate cancer progression is driven by androgen and androgen receptor signaling (3).

In addition to ligands, various coactivators and corepressors contribute to the transcriptional regulation of nuclear receptors (4). Testicular orphan nuclear receptor 4-associated protein 16 (TRA16) was initially identified as a corepressor of TR4 (5). Previous studies have shown that TRA16 promotes non-small cell lung cancer (NSCLC) by activating estrogen receptor beta and inhibiting testicular orphan nuclear receptor 2 (6). While TRA16 overexpression has been implicated in lung carcinogenesis, its role in other cancer types remains largely unexplored.

Recent pan-cancer studies have highlighted the potential to uncover shared cancer characteristics that could inform personalized cancer treatments (7). Advances in next-generation sequencing and single-cell sequencing technologies have led to the generation of massive cancer profiling datasets (8). These datasets have allowed researchers to revisit long-standing questions in cancer biology (9). In this study, we conducted a pan-cancer analysis of TRA16 using data from The Cancer Genome Atlas (TCGA) and the single-cell atlas of human cancers, providing new insights into the role of TRA16 in carcinogenesis.

## Materials and methods

### Pre-processing of single cell datasets

For GSE156625 (liver cancer), the pre-processed integrated dataset was downloaded, and only cells from the tumor atlas were used for this study. A total of 4,756 cells were retained for the final analysis, with cell annotations matching those from the original publication. For GSE182109 (glioma), four samples (GSM5518630, GSM5518631, GSM5518632, GSM5518638) were integrated using canonical correlation analysis (CCA). The FindIntegrationAnchors and IntegrateData functions in Seurat were employed with the top 50 principal component analysis (PCA) dimensions. Cells with detected gene counts between 100 and 7,500 were retained for downstream analysis. Cells with more than 50,000 total UMI counts or with over 20% mitochondrial gene content were filtered out. Nearest neighbors were identified using the FindNeighbors function with the top 20 PCA dimensions, and cell clusters were defined using the FindClusters function with a resolution parameter of 1. Clusters were annotated based on the average expression of classic cell-type-specific markers.

For GSE125449 (liver cancer), two datasets (Set1 and Set2) were used to create Seurat objects, which were then integrated using CCA with the top 50 PCA dimensions. The original cell-type annotations were retained, with “unclassified” and “HPC-like” cells filtered out. The remaining cells were further grouped based on TRA16 UMI levels, and the final analysis included all eligible cells.

### TCGA data mining with Gepia2

To explore the expression pattern and prognostic significance of TRA16 across various cancer types, we conducted a comprehensive re-analysis of TCGA datasets using the GEPIA2 (Gene Expression Profiling Interactive Analysis 2) platform (http://gepia2.cancer-pku.cn) (10). For survival analysis, the Survival Map and Survival Analysis modules were used. Patients were stratified into high and low expression groups based on the median expression level of TRA16. Both overall survival (OS) and disease-free survival (DFS) were assessed. The log-rank test was applied to determine statistical significance, with a significance threshold of p < 0.05. P-values were not adjusted for multiple testing, in line with the default GEPIA2 settings.

### Inference of cell cycle phase from single cell data

Cell cycle scoring from single-cell transcriptomic data was performed using the CellCycleScoring function in Seurat. Each cell was assigned a score based on the expression levels of G2/M and S phase markers. Cell cycle phase (G1, S, or G2/M) was predicted based on these scores. The gene set used for cell cycle scoring, cc.genes.updated.2019, was originally derived from a melanoma study.

### Identification of differentially expressed genes and pathway enrichments

Differentially expressed genes (DEGs) in each cell cluster were identified using the FindAllMarkers function in Seurat, with the parameters test.use = “wilcox”, min.pct = 0.25, and logfc.threshold = 0.25. Genes were further filtered by p-value < 0.05. Pathway enrichment analysis was performed with the R package ClusterProfiler (v4.0.5) using the DEGs as input (11). For Gene Ontology (GO) enrichment analysis, the p-value and q-value cutoffs were set to 0.01 and 0.05, respectively, with p-values corrected using the Benjamini-Hochberg (BH) method. Genes with a log2 fold change greater than 0.5 were included in the analysis, and the top 5 enriched pathways were visualized.

### GSVA

GSVA analysis was conducted using the GSVA package (v1.38.2) in R (12). Hallmark pathways were retrieved from MSigDB (v7.4.1). The normalized data slot from the RNA assay was used as input for GSVA, with “Gaussian” selected as the kernel for non-parametric estimation of the cumulative distribution function of expression levels.

### Cell-cell communication analysis

Cell-cell communication networks were reconstructed using CellChat (13), which predicts cell-cell communication probabilities by integrating gene expression data with curated databases of signaling ligands, receptors, and cofactors based on a mass action model. The communication probability for each signaling pathway was calculated by summarizing all related ligand-receptor interactions. Differences in overall signaling were tested using the paired Wilcoxon test, with a significance cutoff of p < 0.05. Significant ligand-receptor pairs and their communication probabilities between cell groups were visualized using dot plots.

### SCENIC analysis

pySCENIC (v0.11.2) was used to infer potential regulatory transcription factors (TFs) and their target genes in cell clusters (14). pySCENIC identifies TFs and their regulons (TF-target gene sets) by analyzing gene expression correlations across cells, followed by refining the regulons using enriched motif analysis. The activity of each regulon was measured by AUCell scores, where higher AUCell values indicate higher activity and enrichment of the regulon in individual cells.

### Analysis of TRA16 similar genes

TRA16-associated genes were identified using Pearson correlation analysis for each of the 33 TCGA cancer datasets. Genes that were among the top 1,000 TRA16-correlated genes in at least 11 datasets were selected for further analysis. These genes were subjected to pathway enrichment, protein-protein interaction analysis, and transcription factor inference using Metascape with default parameters (15).

### Analysis of mutation patterns

Mutation data for TCGA liver cancer, melanoma, and lung cancer were downloaded from the Genomic Data Commons (GDC) via TCGAbiolinks (16). Query filters included data category (“Simple Nucleotide Variation”), data type (“Masked Somatic Mutation”), and experimental strategy (“WXS”). RNA expression data for patient stratification were also downloaded using TCGAbiolinks, with filters for data category (“Gene Expression”), data type (“Gene Expression Quantification”), and experimental strategy (“RNA-Seq”). Oncoprint plots were generated to visualize the top mutated genes in each patient group.

### Analysis of stromal and immune features

To assess the tumor microenvironment (TME) composition, stromal, immune, and ESTIMATE scores were computed using the tidyestimate package (v1.1.1), which implements the ESTIMATE algorithm based on single-sample gene set enrichment analysis (ssGSEA) of predefined stromal and immune gene signatures. Expression data were formatted to meet the requirements of the ESTIMATE algorithm, with HGNC gene symbols as row identifiers and sample IDs as columns.

For correlation analysis, TRA16 expression values were extracted across all samples. Spearman correlation coefficients were calculated between TRA16 expression and stromal, immune, and ESTIMATE scores. Samples were further stratified into “TRA16-high” and “TRA16-low” groups using the median expression value as a cutoff. Expression levels of canonical immune checkpoint genes PD-L1 (CD274) and CTLA4 were compared between TRA16-high and TRA16-low groups using the Wilcoxon rank-sum test.

### Immunohistochemical staining and interpreting

The IHC-validation cohort consisted of consecutive patients diagnosed with ovarian clear cell carcinoma (OCCC), who underwent surgical resection from 2016 to 2023 at Peking Union Medical College Hospital. Tissue microarrays were constructed from formalin-fixed, paraffin-embedded tissues. Both primary tumors and normal endometrium were included. 4-μm tissue sections were deparaffinized and rehydrated. Antigen retrieval was processed at pH 6 for 20 minutes. The IHC staining process was performed with DAKO Autostainer Link 48 using TRA16 polyclonal antibody (HPA042054, Sigma-Aldrich) in 1:200 dilution.

As described in previous studies (17, 18), each core was evaluated by composite scores (ranging from 0 to 12) based on both intensity and percentage of positive cells. Cases with composite scores ≥ 4 points were considered positive, while others were considered negative.

### Statistical analysis

GraphPad Prism software Version 8.0 (GraphPad, San Diego, CA) was used to analyze and visualize the immunostaining data. All other statistical analyses were performed in R unless otherwise specified. T-tests were used to compare gene expression between 2D and organoid cultures using TPM matrix. For count matrix (GSE274674), edgeR method was employed. Wilcoxon tests were applied to compare mutation numbers between patient groups, to identify cluster-specific markers, and to determine differentially expressed genes between TRA16-positive and TRA16-negative cells, with a significance threshold of p < 0.05. For survival analysis, log-rank tests were employed to compare survival curves between patient groups. Pearson correlation analysis was used to identify genes correlated with TRA16 expression. For Gene Ontology (GO) enrichment analysis, p-values and q-values were set at 0.05, with p-values corrected using the Benjamini-Hochberg method.

## Results

### Expression and prognostic significance of TRA16 across cancer types

**Figure 1:**
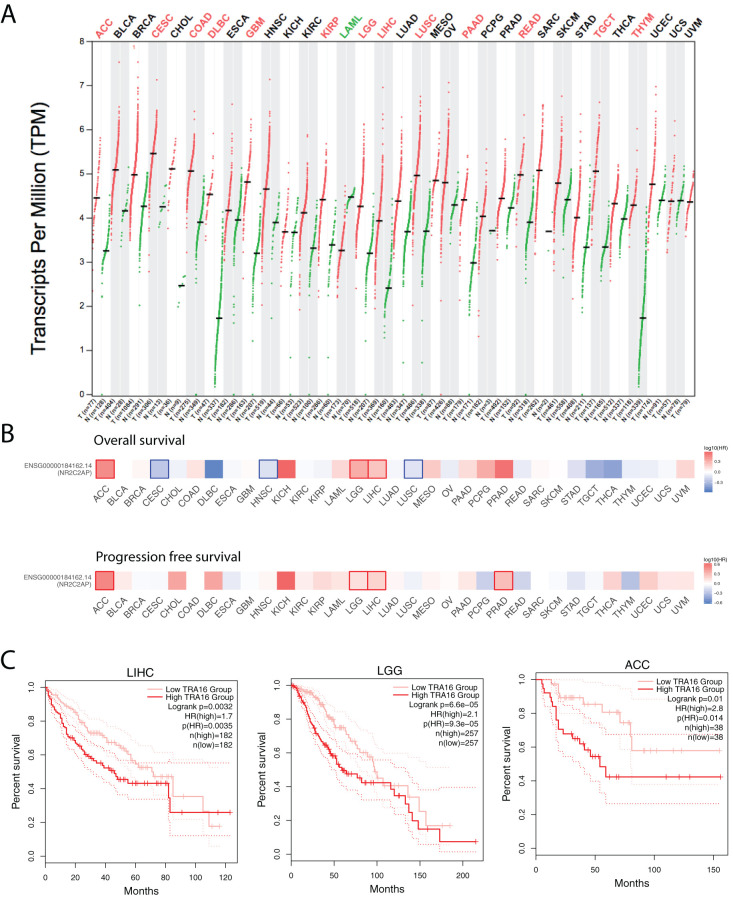
Expression and prognostic significance of TRA16 across cancer types. **(A)** Transcripts per Million Reads (TPM) for TRA16 shown in log2 scale for tumor tissues (T) and normal tissues (N) for the TCGA datasets. Red indicates the cancer types where TRA16 is significantly upregulated in tumor tissues compared with normal tissues. **(B)** TCGA cancer cohorts (33 different types) were each stratified by TRA16 RNA expression. Survival analysis was performed with overall survival (top panel) and progression free survival (bottom panel). Heatmap was used to present results of survival analysis. Hazard ratio was shown in blue to red color scale. Red squares indicate significant prognostic factor when high TRA16 correlates with bad prognosis. Blue squares indicate significant prognostic factor when high TRA16 correlates with good prognosis. **(C)** Survival curves for TCGA liver cancer cohort (LIHC), low grade glioma (LGG) and Adrenocortical Carcinoma (ACC). Red curve presents patients with high TRA16 expression and pink curve presents patients with low TRA16 expression.

To investigate the functional role of TRA16 in human cancers, we analyzed TRA16 expression levels in both cancer and normal tissues using the TCGA datasets. Differential expression data for 28 cancer types were retrieved from GEPIA2. TRA16 was significantly upregulated in 13 cancer types, including adrenocortical carcinoma (ACC), cervical squamous cell carcinoma (CESC), colon adenocarcinoma (COAD), diffuse large B-cell lymphoma (DLBC), glioblastoma multiforme (GBM), kidney renal papillary cell carcinoma (KIRP), lower-grade glioma (LGG), liver hepatocellular carcinoma (LIHC), lung squamous cell carcinoma (LUSC), pancreatic adenocarcinoma (PAAD), rectum adenocarcinoma (READ), testicular germ cell tumors (TGCT), and thymoma (THYM). In contrast, TRA16 was significantly downregulated in only one cancer type: acute myeloid leukemia (LAML) (**Figure 1A**).

To assess the prognostic potential of TRA16, we analyzed overall survival (OS) and progression-free survival (PFS) across all cancer types by stratifying patients based on median TRA16 RNA expression (**Figure 1B**). TRA16 emerged as a significant prognostic marker for both OS and PFS in three cancer types: ACC, LGG, and LIHC. Survival curves comparing patient groups with high and low TRA16 expression were generated for these cancers, with log-rank p-values of 0.0032 (LIHC), 6.6e-05 (LGG), and 0.01 (ACC), respectively, and confidence intervals were provided (**Figure 1C**).

### Single cell analysis of TRA16 in liver cancer

**Figure 2:**
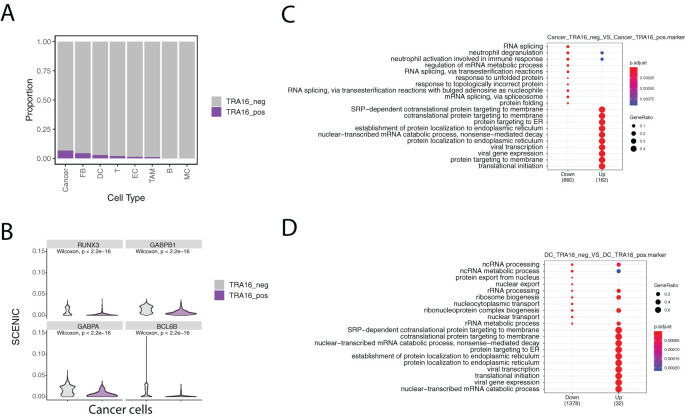
Single cell analysis of TRA16 in liver cancer. **(A)** Stacked bar plot visualizing the percentage of TRA16 positive cells in each cell type. **(B)** Violin plot showing top transcription factors with tumor suppressor roles. **(C)** Dot plot summarizing the top 10 enriched terms for genes down-regulated in TRA16 positive cancer cells and genes up-regulated in TRA16 negative cancer cells. **(D)** Dot plot summarizing the top 10 enriched terms for genes down-regulated in TRA16 positive dendritic cells and genes up-regulated in TRA16 negative dendritic cells.

Given the high incidence of liver cancer, we analyzed TRA16 expression in a previously published single-cell atlas of liver cancer (GSE156625). Cell types in the atlas were annotated using the original study’s labels, with some re-grouping for clarity. For instance, subclusters of tumor-associated macrophages (TAMs) were merged into a single TAM group, and fetal B cells (B cells with embryonic characteristics) were combined with mature B cells. In total, eight major cell types were retained: cancer cells, dendritic cells (DCs), fibroblasts (FBs), endothelial cells (ECs), B cells (B), T cells (T), TAMs, and monocytes (MCs). TRA16-positive cells were identified in all major cell types except monocytes and B cells (**Figure 2A**).

The highest percentages of TRA16-positive cells were observed in cancer cells (6.7%), fibroblasts (4.3%), and dendritic cells (2.8%). To further investigate transcription factor (TF) activity, we used the SCENIC algorithm to compare TRA16-positive and TRA16-negative cancer cells. Among the top 10 differentially active TFs, eight showed decreased activity in TRA16-positive cancer cells. Notably, four of these TFs—RUNX3, GABPB1, GABPA, and BCL6B—are known tumor suppressors (**Figure 2B**).

Cells were stratified based on TRA16 expression, with TRA16-positive cells defined as those with at least one UMI detected for TRA16, and TRA16-negative cells as those without any detected UMI for TRA16. Differentially expressed genes between the two groups were identified using the Wilcoxon test. These differentially expressed genes were then subjected to Gene Ontology (GO) enrichment analysis using an over-representation method. Interestingly, genes upregulated in TRA16-positive cancer cells were enriched for several key biological processes, including SRP-dependent cotranslational protein targeting to the membrane, cotranslational protein targeting to the membrane, protein targeting to the endoplasmic reticulum (ER), establishment of protein localization to the ER, protein targeting to the membrane, and translational initiation (**Figure 2C**). Notably, genes upregulated in TRA16-positive dendritic cells were also enriched for these same processes, mirroring the enrichment observed in cancer cells (**Figure 2D**).

### Landscape of intercellular communication with or without TRA16 expression

**Figure 3:**
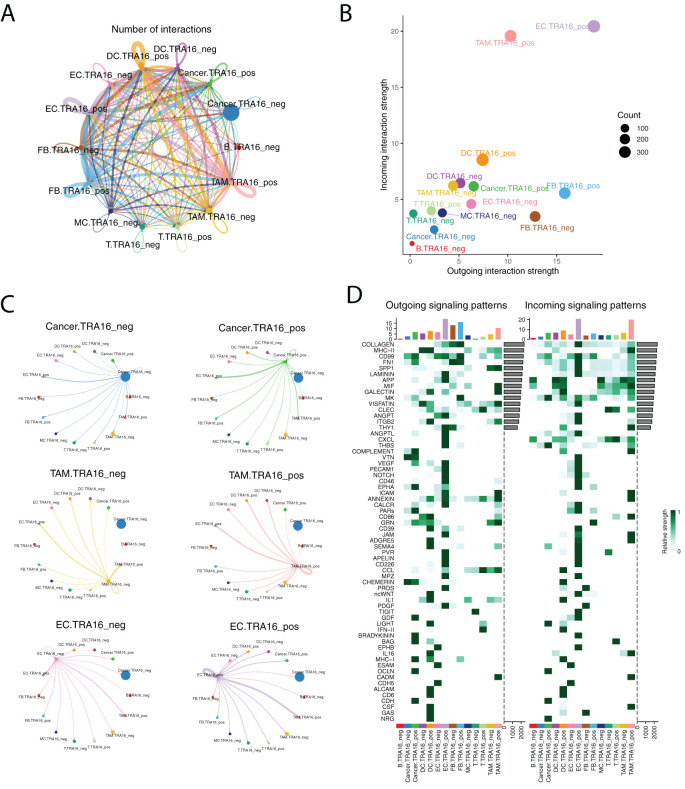
Landscape of intercellular communication with or without TRA16 expression. **(A)** The number of interactions was visualized using circle plot. The thickness of lines is in proportion to the number of interactions between two cell types. Each cell type is color coded with one distinct color. **(B)** The incoming interaction strength and outgoing interaction strength were shown as scatter plots for each cell type. **(C)** Separate circle plot was used to present outgoing signaling from TRA16 negative cancer cells, TRA16 positive cancer cells, TRA16 negative TAM, TRA16 positive TAM, TRA16 negative EC and TRA16 positive EC. The dot size is proportional to the number of cells of corresponding cell type and the thickness of lines is proportional to the number of interactions between two cell types. **(D)** The outgoing signaling patterns (left panel) and the incoming signaling patterns (right panel) were visualized with heatmaps. Each row indicates one signaling pathway and each column indicates one cell type. The top bar plot shows the assembled outgoing or incoming signaling for each cell type. The side bar visualizes the aggregated outgoing or incoming signaling of each pathway.

Next, we investigated whether TRA16 expression influences differential cell-cell communication patterns. Using CellChat and single-cell transcriptomic data, we constructed a complex intercellular communication network. Notably, TRA16 expression appeared to enhance autocrine signaling in several cell types, including dendritic cells, tumor-associated macrophages, endothelial cells, and fibroblasts (**Figure 3A**). TRA16 expression also increased the overall strength of both incoming and outgoing intercellular communication (**Figure 3B**). Among these, TRA16-positive endothelial cells exhibited the highest signaling strength in both directions, likely reflecting an adaptive mechanism involved in tumor angiogenesis.

At the individual cell type level, TRA16 expression induced distinct perturbations in communication patterns (**Figure 3C**). TRA16-positive cancer cells showed increased interaction with TRA16-positive endothelial cells, while TRA16-positive tumor-associated macrophages secreted more autocrine signals. TRA16-positive endothelial cells demonstrated stronger signaling both to themselves and to TRA16-positive tumor-associated macrophages.

We further analyzed the specific outgoing and incoming signaling pathways that differed between TRA16-positive and TRA16-negative cells (**Figure 3D**). TRA16-positive cancer cells exhibited increased outgoing signaling via COMPLEMENT, VEGF, EPHA, PARs, GDF, BRADYKININ, BAG, MHC-I, OCLN, and CDH pathways, while receiving more incoming signaling through MK, THBS, GDF, LIGHT, OCLN, CDH, and NRG pathways. For endothelial cells, top 10 elevated outgoing signaling pathways included LAMININ, APP, THY1, ANGPTL, CXCL, THBS, VEGF, PECAM1, NOTCH, CD46, while top 10 enhanced incoming signaling pathways were COLLAGEN, CD99, FN1, SPP1, LAMININ, VISFATIN, ANGPT, ITGB2, VTN, VEGF.

Using an independent single-cell atlas of liver cancer (GSE125449), we confirmed that TRA16 was predominantly expressed by malignant cells (**Supplementary Figure 1A**). Consistent with previous observations, TRA16 expression increased the overall strength of both incoming and outgoing signaling across most cell types (**Supplementary Figure 1B**), though the specific pathways perturbed were not completely identical (**Supplementary Figure 1C**).

Next, we analyzed a single-cell atlas of low-grade glioma (LGG) using a previously published dataset. The immune microenvironment and stromal composition of LGG were markedly different from liver cancer, with B cells and fibroblasts being almost absent. Notably, the highest percentage of TRA16-positive cells was found in cycling glioma cancer cells (**Supplementary Figure 2A**). Similar to liver cancer, TRA16 expression enhanced the overall interaction strength of both incoming and outgoing signals in LGG (**Supplementary Figures 2B, C**).

### TRA16-associated genes are implemented in cell cycle regulation

As confirmed by our pan-cancer analysis of TCGA datasets, TRA16 is upregulated in cancer tissues compared to normal tissues. Next, we sought to identify genes co-regulated with TRA16. To do this, we employed a straightforward approach to detect similar genes. For each of the 33 TCGA cancer datasets, we identified the top 1000 genes correlated with TRA16 expression using Pearson correlation analysis. Genes that ranked among the top 1000 in at least 11 datasets were selected for downstream analysis. In total, 595 genes were subjected to pathway enrichment and transcription factor enrichment analyses.

**Figure 4:**
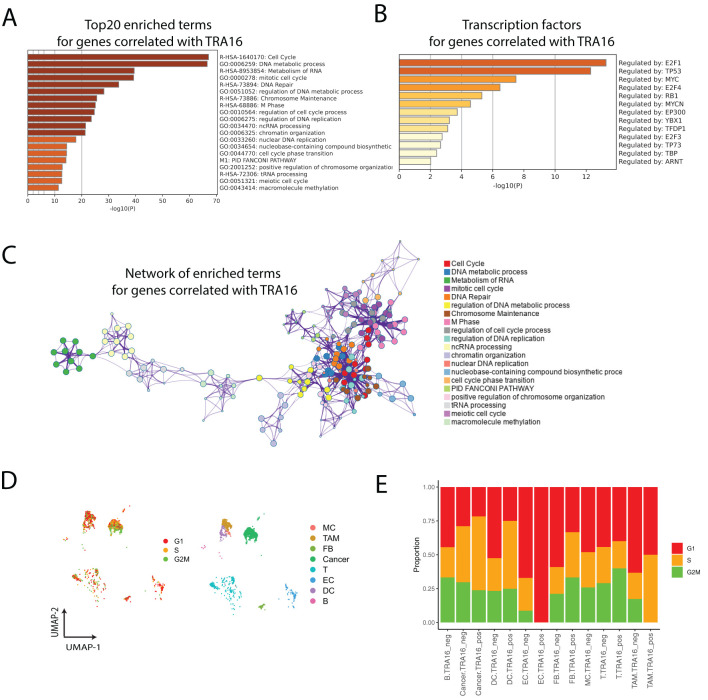
TRA16-associated genes are implemented in cell cycle regulation. **(A)** Top 20 enriched terms for genes correlated with TRA16. **(B)** Transcription factors enriched for genes correlated with TRA16. **(C)** The enriched terms for genes correlated with TRA16 visualized as a network. **(D)** Each cell is color coded by the cell cycle phase inferred from single cell transcriptomic data. **(E)** Stacked bar plot visualizing the relative distributions of cell cycle phases inferred in **(D)**.

Interestingly, the top 20 pathways enriched for TRA16-associated genes were predominantly related to the cell cycle or cell cycling processes (**Figure 4A**). The top five enriched transcription factors were E2F1, TP53, MYC, E2F4, and RB1, consistent with cell cycle regulation (**Figure 4B**). Notably, three members of the E2F family—E2F1, E2F4, and E2F3—were among the enriched transcription factors. Network visualization of the enriched terms revealed a strong representation of cell cycle-related pathways among the TRA16-associated genes (**Figure 4C**).

To further explore the role of TRA16 in cell cycle regulation at the single-cell level, we inferred the cell cycle phase (G1, S, G2M) for individual cells in the liver cancer dataset (**Figure 4D**). Consistent with the involvement of TRA16-associated genes in the cell cycle, we observed an increased percentage of cells in the S or G2M phases among TRA16-positive cells across multiple cell types, including dendritic cells, fibroblasts, tumor-associated macrophages, and cancer cells (**Figure 4E**).

### TRA16 is upregulated in liver cancer organoids

**Figure 5:**
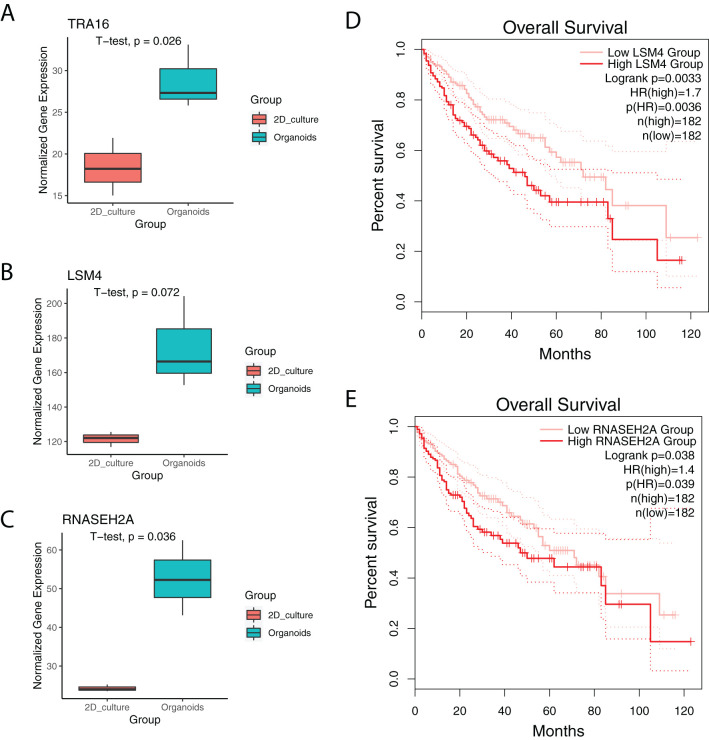
TRA16 is upregulated in liver cancer organoids. **(A)** Boxplot showing the expression of TRA16 in 2D cultures or organoid cultures of Hep G2. **(B)** Boxplot showing the expression of LSM4 in 2D cultures or organoid cultures of Hep G2. **(C)** Boxplot showing the expression of RNASEH2A in 2D cultures or organoid cultures of Hep G2. **(D)** Survival curve for LSM4 low expression liver cancer patients (pink) and LSM4 high expression liver cancer patients (red). Dashed lines indicate 95% confidence interval. **(E)** Survival curve for LSM4 low expression liver cancer patients (pink) and LSM4 high expression liver cancer patients (red). Dashed lines indicate 95% confidence interval.

In a previous study, we profiled HepG2 liver cancer cells cultured as either 2D adherent cells or 3D organoids (19). Notably, TRA16 was significantly upregulated in liver cancer organoids (**Figure 5A**). TRA16 upregulation in 3D culture was further observed in cervical cancer cell line SiHa (**Supplementary Figure 3A**). Similarly, the expression levels of LSM4 (p = 0.072) and RNASEH2A (p = 0.036) were also elevated in liver cancer organoids (**Figures 5B, C**). Pan-cancer analysis using TCGA tumor samples revealed a significant positive correlation between TRA16 and LSM4 (R = 0.63), as well as TRA16 and RNASEH2A (R = 0.6) (**Supplementary Figures 3C–E**). To further investigate the clinical relevance, we stratified patients based on the median expression levels of LSM4 and RNASEH2A into low-expression and high-expression groups. Survival analysis revealed significantly different survival curves for both LSM4 (**Figure 5D**) and RNASEH2A (**Figure 5E**), suggesting their potential prognostic value.

### Mutational landscape of liver cancer with high TRA16

Given the potential role of TRA16 in liver cancer, we next investigated whether liver cancers with high TRA16 expression exhibit distinct mutation patterns compared to those with low TRA16 expression. TCGA liver cancer patients with RNA expression data (n = 415) were stratified into high and low expression groups based on the median TRA16 expression. Among these patients, 371 had whole exome sequencing data available.

**Figure 6:**
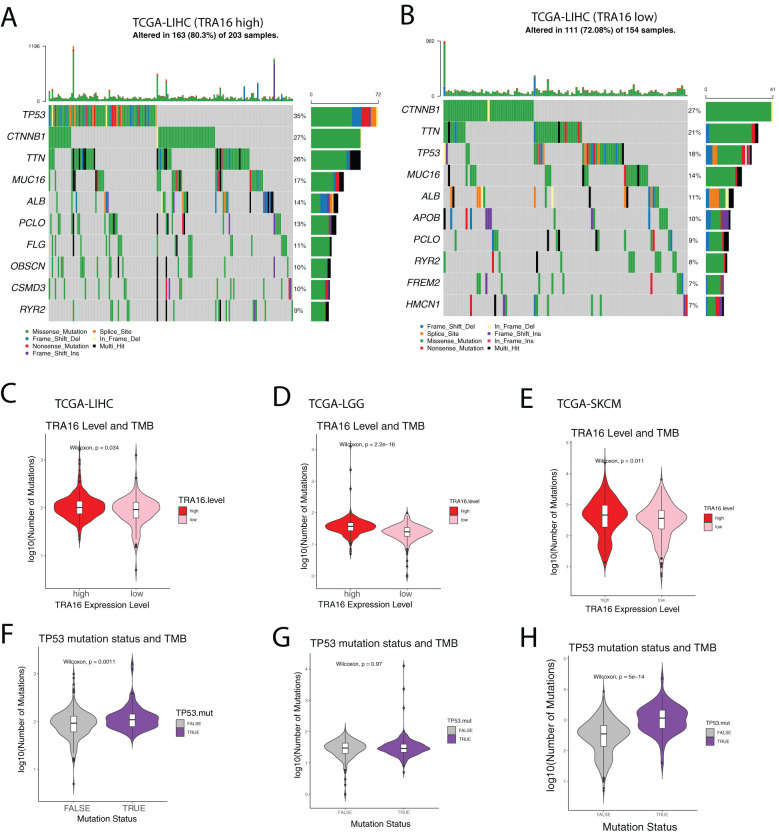
Mutational landscape of liver cancer with high TRA16. **(A)** Oncoprint plot showing the top 10 most mutated genes in TCGA-LIHC cohort with high TRA16 expression. Different mutation types were color coded. **(B)** Oncoprint plot showing the top 10 most mutated genes in TCGA-LIHC cohort with low TRA16 expression. Different mutation types were color coded. **(C)** Violin plot displaying the number of mutations in TRA16 high expression (red) and low expression (pink) patients for TCGA-LIHC cohort. Within the Violin, a boxplot was used to present the quantile information of the data. **(D)** Violin plot displaying the number of mutations in TRA16 high expression (red) and low expression (pink) patients for TCGA-LGG cohort. Within the Violin, a boxplot was used to present the quantile information of the data. **(E)** Violin plot displaying the number of mutations in TRA16 high expression (red) and low expression (pink) patients for TCGA-SKCM cohort. Within the Violin, a boxplot was used to present the quantile information of the data. **(F)** Violin plot displaying the number of mutations in TP53 wild type (grey) and TP53 mutated (purple) patients for TCGA-LIHC cohort. Within the Violin, a boxplot was used to present the quantile information of the data. **(G)** Violin plot displaying the number of mutations in TP53 wild type (grey) and TP53 mutated (purple) patients for TCGA-LGG cohort. Within the Violin, a boxplot was used to present the quantile information of the data. **(H)** Violin plot displaying the number of mutations in TP53 wild type (grey) and TP53 mutated (purple) patients for TCGA-SKCM cohort. Within the Violin, a boxplot was used to present the quantile information of the data.

In the TRA16 high-expression group, the top 10 most frequently mutated genes were TP53, CTNNB1, TTN, MUC16, ALB, PCLO, FLG, OBSCN, CSMD3, and RYR2 (**Figure 6A**). In contrast, the top 10 mutated genes in the TRA16 low-expression group were CTNNB1, TTN, TP53, MUC16, ALB, APOB, PCLO, RYR2, FREM2, and HMCN1 (**Figure 6B**). Notably, the frequency of TP53 mutations increased from 18% in the low-expression group to 35% in the high-expression group.

Given the established correlation between TP53 mutations, mutation burden, and response to immunotherapy, we further quantified the number of mutations in each group (**Figures 6C–H**). Patients with high TRA16 expression exhibited a significantly higher mutation burden, suggesting a potential link between TRA16 expression and mutation-driven oncogenesis.

### TRA16 expression inversely correlates with stromal and immune features

**Figure 7:**
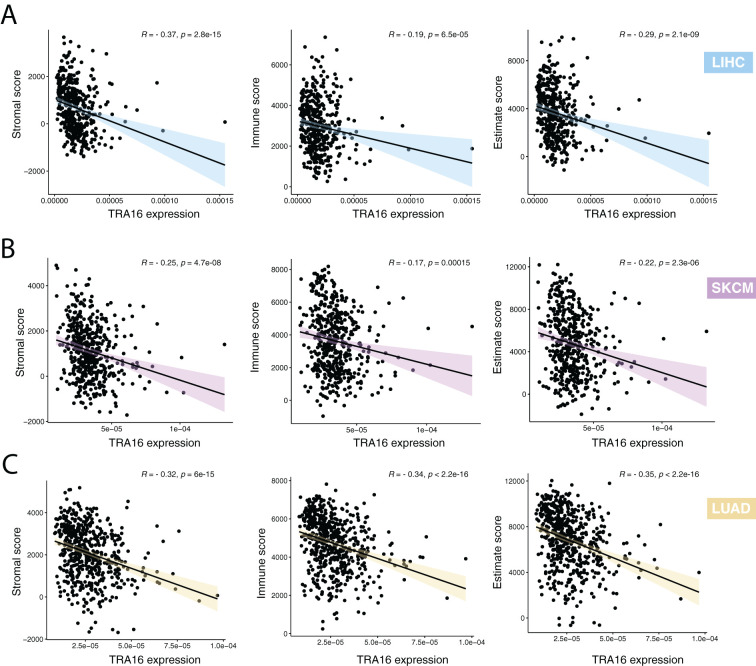
High TRA16 expression negatively correlates with stromal and immune features. **(A)** The correlations between TRA16 RNA expression and stromal, immune and estimate scores were shown for LIHC. **(B)** The correlations between TRA16 RNA expression and stromal, immune and estimate scores were shown for SKCM. **(C)** The correlations between TRA16 RNA expression and stromal, immune and estimate scores were shown for LUAD.

Stromal and immune cells are two key components of the tumor microenvironment. We evaluated the relationship between the expression of TRA16 and the tumor microenvironment composition using ESTIMATE-derived scores (non-tumor scores). Spearman correlation analysis revealed a significant inverse correlation between TRA16 expression and stromal, immune, and ESTIMATE scores (**Figures 7A–C**). These findings suggest that higher TRA16 expression is associated with reduced infiltration of both stromal and immune components, indicating that TRA16 may be preferentially expressed in tumor-cell–dominant regions with a less prominent microenvironmental presence.

To further explore the immunological context of TRA16 expression, we stratified samples into TRA16-high and TRA16-low groups based on the median expression value and compared the expression levels of immune checkpoint genes. Notably, PD-L1 (CD274) expression was significantly down-regulated in the TRA16-high group (**Supplementary Figure 4**), suggesting that tumors with higher TRA16 expression may exhibit enhanced immunotherapy response. In contrast, CTLA4 expression showed less consistent differences between the two groups, with no statistically significant trend observed.

### Expression of TRA16 at protein level

We further performed immunohistochemical staining to investigate TRA16 protein expression level in cancer and non-malignant tissues. Ovarian clear cell carcinoma (OCCC) is a specific histological subtype of ovarian cancer with several mutational similarities with KIRC (20). Due to limited access to normal ovarian samples and the recognition that OCCC originates from endometriosis (21), we compared OCCC (n = 208) and normal endometrium (n = 187) with immunohistochemistry (**Supplementary Figures 5A–D**). It was discovered that cancer tissues from OCCC patients were significantly more likely to exhibit positive expression of TRA16 (78.3% vs 21.7%, p<0.001) compared with normal endometrium tissues (**Supplementary Figure 5E**). As for paired samples of OCCC tumor and normal endometrium (n=159 pairs), OCCC tumors showed higher composite scores (Wilcoxon test, p<0.0001) than normal endometrium (**Supplementary Figure 5F**). However, there was no significant difference in survival between OCCC patients with positive TRA16 expression and those with negative TRA16 expression (**Supplementary Figure 5G**). Positive staining of TRA16 was also observed in various cancer types in the Human Protein Atlas (**Supplementary Figure 6**). Considering the current limited protein level characterization of TRA16 in cancer and paired normal tissues, future studies combining large scale biobank of tissue microarrays and high throughput *in situ* protein level profiling might provide a more comprehensive pan-cancer view of TRA16 protein expression and enable a comparison between RNA and protein expression.

## Discussion

**Figure 8:**
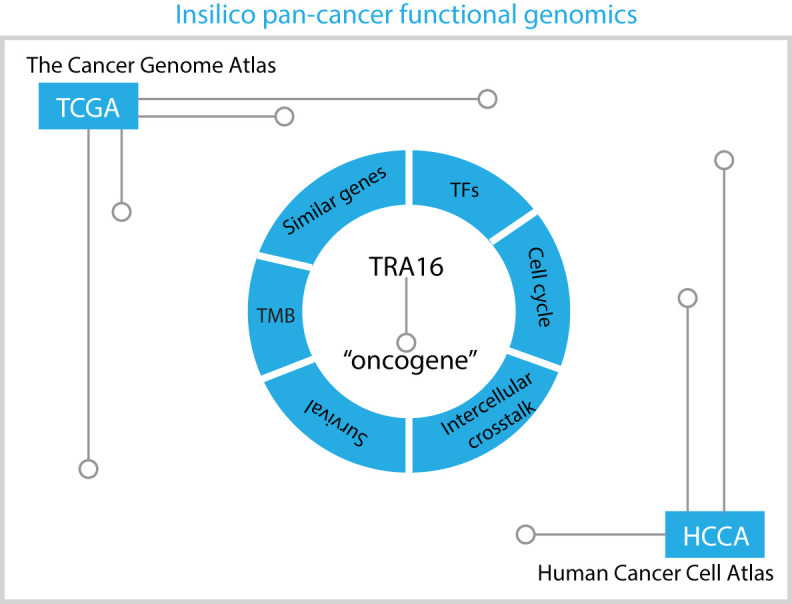
Schematic summary of in silico pan-cancer analysis. The potential role of TRA16 as an oncogene revealed by in silico analysis of transcription factors, cell cycle, similar genes, intercellular crosstalk, TMB and survival significance.

Over the past decade, massive datasets profiling cancer patients at both the bulk tissue and single-cell levels have been generated. These datasets offer a valuable resource for pan-cancer analysis, particularly for investigating genes of interest. In our study, we adopted an in silico pan-cancer functional genomic approach to investigate the role of TRA16 in cancer (**Figure 8**). Since its discovery in 2003, very few publications have referenced TRA16 in PubMed. Earlier studies demonstrated that TRA16 could be detected in 88.64% of non-small cell lung cancers (NSCLC) but was absent in normal lung tissue and benign lung tumors (22). A subsequent study further revealed that TRA16 promotes cancer cell growth by activating the estrogen receptor beta (ERβ) pathway (6).

Cancer development and progression are driven by a complex network of intercellular communication (23). Notably, cancer cells rely on endothelial cells to proliferate and form new blood vessels. These newly formed vessels not only supply nutrients to the tumor but also serve as gateways for immune cells to infiltrate and surveil the tumor tissue. Our discovery that TRA16-positive cancer cells and endothelial cells exhibit more active intercellular communication highlights TRA16 as a potential novel target for anti-angiogenesis therapy by disrupting cellular interactions within the tumor microenvironment.

Our pan-cancer analysis revealed that genes co-expressed with TRA16 are regulated by E2F1, TP53, MYC, E2F4, and RB1. Both the E2F family of transcription factors and MYC are crucial regulators of cell cycle progression (24, 25). RB1, the first tumor suppressor gene ever discovered, functions as an upstream regulator of E2F1 (26). This aligns with the hypothesis that TRA16 may play a role in cell cycle regulation.

Two TRA16-associated genes, LSM4 and RNASEH2A, were found to be upregulated in organoid cultures of HepG2 cells. LSM4 has been implicated in the progression of pancreatic cancer (27), breast cancer (28) and hepatocellular carcinoma (29). RNASEH2A, a member of the RNase HII family, plays critical roles in the invasiveness and chemoresistance of breast cancer (30), proliferation and apoptosis in glioma (31), DNA damage response and cell viability of T cell leukemia (32). The co-expression network of RNASEH2A is involved in DNA replication, DNA damage response, and cell cycle regulation (33).

Our study also highlights the role of TRA16 in liver cancer. The risk of liver cancer significantly increases in patients with viral hepatitis (34). Chronic infection with hepatitis B or C viruses leads to increased hepatocyte apoptosis and regeneration. When key genes in the DNA damage repair pathway are mutated, resulting in loss of function, cells with DNA damage become resistant to apoptosis (35). The elevated expression of TRA16 in cells with more somatic mutations may represent an adaptive mechanism that supports cell survival and sustained proliferation. This mechanism likely helps cancer cells remain viable and maintain fitness in response to environmental stress.

The potential link between TP53 mutations and TRA16 expression uncovered in our study could be leveraged to develop novel therapeutics for patients with TP53-mutated liver cancer. TP53, a frequently mutated tumor suppressor gene, encodes a protein that plays a crucial role in the DNA damage response and apoptosis. Despite its significance in cancer, TP53 has remained a challenging target for drug development (36). Future studies should explore whether targeting TRA16 could offer an effective alternative strategy for treating TP53-mutated liver cancer patients.

Immune checkpoint blockade (ICB) has revolutionized cancer treatment over the past decade. Clinical studies have shown that patients with a high tumor mutation burden (TMB) respond better to ICB therapy (37). Our finding that TRA16-upregulated cancers may harbor elevated TMB suggests that ICB could be a viable treatment option for this subtype of liver cancer. Furthermore, it might be of interest to explore the usefulness of TRA16 expression in patient stratification for checkpoint inhibitor therapy considering the connection of TP53 mutations and response to immune checkpoint inhibition (38).

The potential role of TRA16 in carcinogenesis is not limited to liver cancer. TRA16 also appears to be a significant prognostic marker in low-grade glioma and adrenocortical carcinoma. Additionally, our results reveal that the association between TRA16 and TMB extends to other cancers, including melanoma, lung cancer, and colon cancer—cancers that are often characterized by high TMB (39). The link between TRA16 and TMB may be either dependent on or independent of TP53 mutations. Future studies are merited to further explore whether TRA16 alone or in combination with other biomarkers can be used to improve the accuracy of cancer patient stratification (40).

However, our study has some limitations. First, while our analysis of large datasets provides valuable insights, it is largely correlative, and correlation does not imply causation. Well-designed *in vitro* and *in vivo* experiments are necessary to determine the effects of TRA16 gain-of-function and loss-of-function mutations (41). Second, dropout events are common in single-cell datasets (42), which can affect the accuracy of grouping cells based on UMI counts. Some cells classified as TRA16-negative may in fact have low TRA16 expression.

In conclusion, we rigorously analyzed publicly available datasets profiling human cancers at both the bulk and single-cell levels to investigate the function of TRA16. Our findings suggest that TRA16 is a potential oncogene and may serve as a master regulator of carcinogenesis.

## Data Availability

The following single-cell datasets were downloaded from GEO: liver cancer cell atlas [GSE156625 (43), GSE125449 (44)], glioma cell atlas [GSE182109 (45)]. Datasets for integrative analysis of transcriptomes and exomes were obtained via TCGAbiolinks, including TCGA-LIHC, TCGA-LGG, and TCGA-SKCM. Processed RNA-seq data for HepG2 adherent and organoid cultures were accessed from Zenodo (doi: 10.5281/zenodo.6481921) (19). RNA-seq dataset of cervical cancer cell line was obtained from GEO [GSE270674 (46)].
