# Supplementary material for: Pan-cancer analysis reveals TRA16 as a master regulator of human carcinogenesis
Source: Front Oncol. 2025 May 13;15:1543419. doi: 10.3389/fonc.2025.1543419 (PMC12106028; doi:10.3389/fonc.2025.1543419)
Supplement: Supplementary file 1 [file DataSheet1.pdf]

Supplementary Figure 1

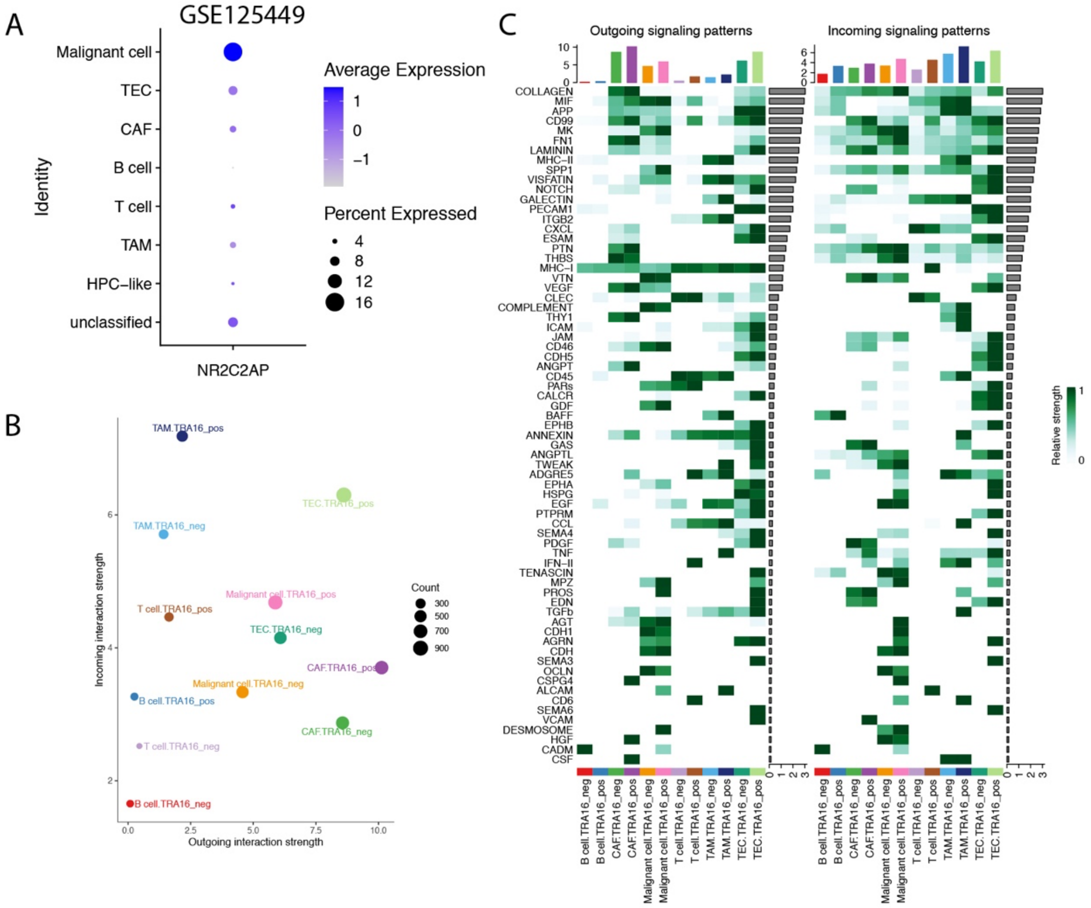

Supplementary Figure 1. Analysis of TRA16 in single cell atlas of liver cancer (GSE125449).

(A) The expression of TRA16 was visualized with dotplot, with the diameter of dot corresponding to the percent of cells expressing TRA16 and the intensity of dot indicating the average expression level of TRA16.

(B) The incoming interaction strength and outgoing interaction strength were shown as scatter plots for each cell type, stratified by TRA16 expression level.

(C) The outgoing signaling patterns (left panel) and the incoming signaling patterns (right panel) were visualized with heatmaps. Each row indicates one signaling pathway and each column indicates one cell type. The top bar plot shows the assembled outgoing or incoming signaling for each cell type. The side bar visualizes the aggregated outgoing or incoming signaling of each pathway.

Supplementary Figure 2

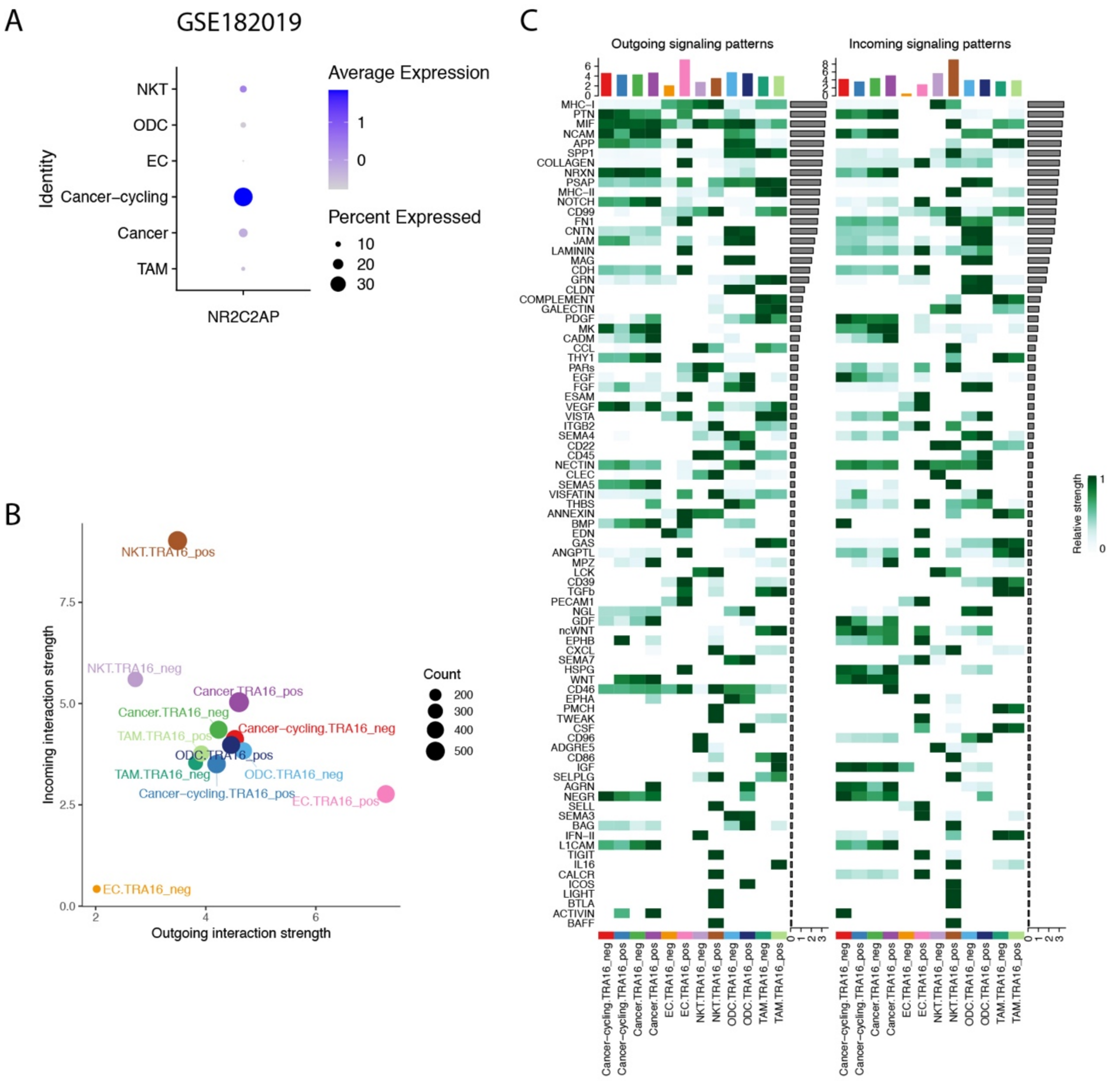

Supplementary Figure 2. Analysis of TRA16 in single cell atlas of low grade glioma (GSE182019).

(A) The expression of TRA16 was visualized with dotplot, with the diameter of dot corresponding to the percent of cells expressing TRA16 and the intensity of dot indicating the average expression level of TRA16.

(B) The incoming interaction strength and outgoing interaction strength were shown as scatter plots for each cell type, stratified by TRA16 expression level.

(C) The outgoing signaling patterns (left panel) and the incoming signaling patterns (right panel) were visualized with heatmaps. Each row indicates one signaling pathway and each column indicates one cell type. The top bar plot shows the assembled outgoing or incoming signaling for each cell type. The side bar visualizes the aggregated outgoing or incoming signaling of each pathway.

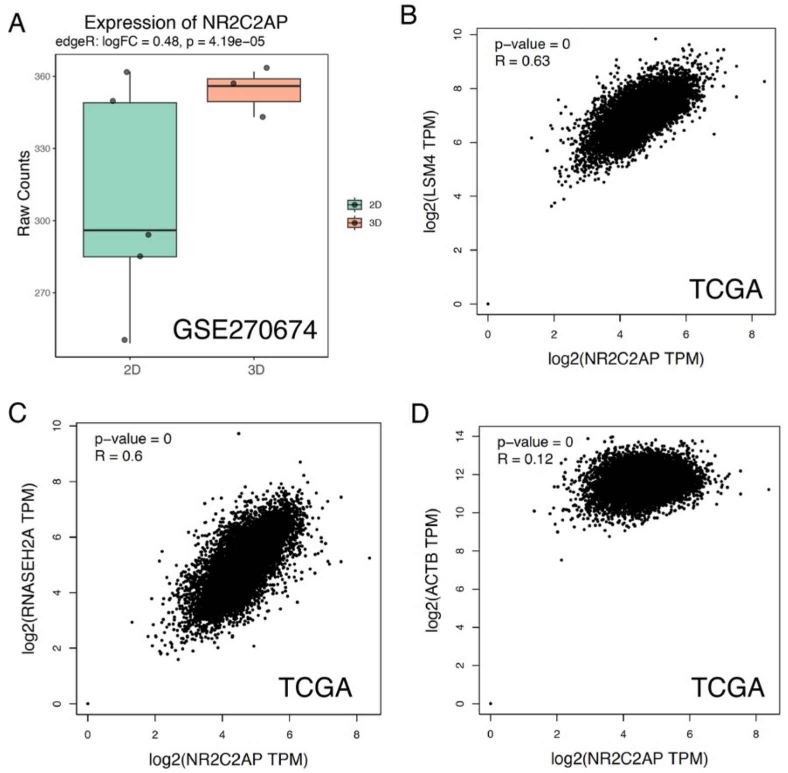

Supplementary Figure 3. Validation of correlation and differential expression using additional datasets.
The differential expression of NR2C2AP between 2D and 3D culture condition was validated in GSE270674 (A). The correlation between NR2C2AP (TRA16) and LSM4 (B) or RNASEH2A (C) or ACTB (D) was analyzed using TCGA dataset.

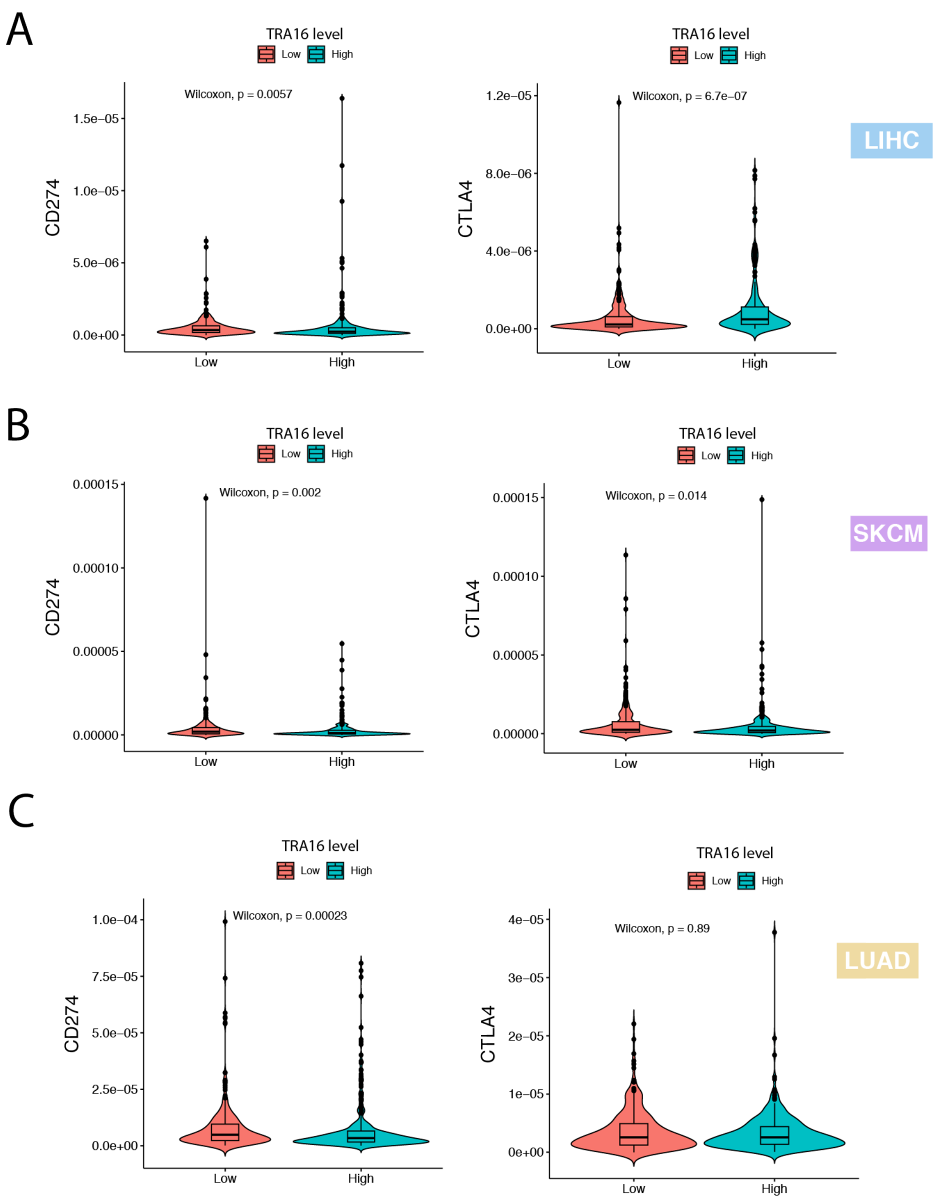

Supplementary Figure 4. The differential expression of CD274 and CTLA4 in TRA16 low and TRA16 high tumors.
The differential expression of CD274 and CTLA4 in TRA16 low and TRA16 high tumors using TCGA-LIHC (A), TCGA-SKCM (B) and TCGA-LUAD (C).

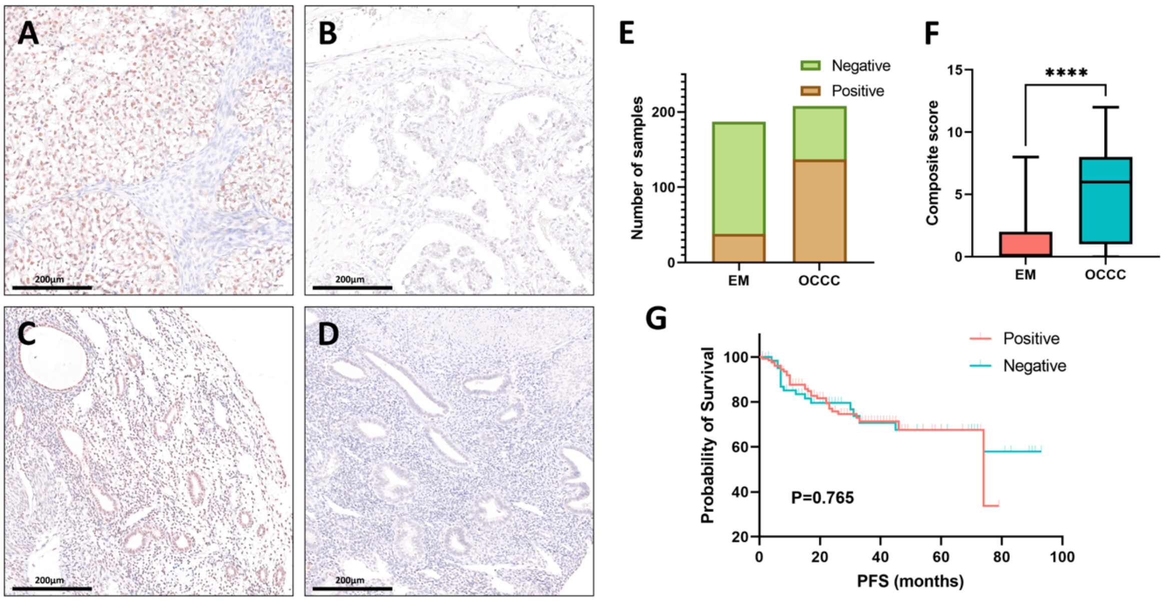

Supplementary Figure 5. Ovarian cancer showed upregulated expression of TRA16 at protein level. (A) OCCC with positive staining of TRA16. (B) OCCC shows negative staining of TRA16. (C) Positive expression of TRA16 in EM. (D) Negative expression of TRA16 in EM. (E) The clustered bar shows TRA16 IHC expression of OCCC and EM. (F) Boxplot shows the difference in composite score of TRA16 expression between OCCC and EM (Wilcoxon test, $p<0.0001$). (G) Survival curve for TRA16 positive patients and TRA16 negative patients. OCCC, ovarian clear cell carcinoma; EM, endometrium. magnification 20x.

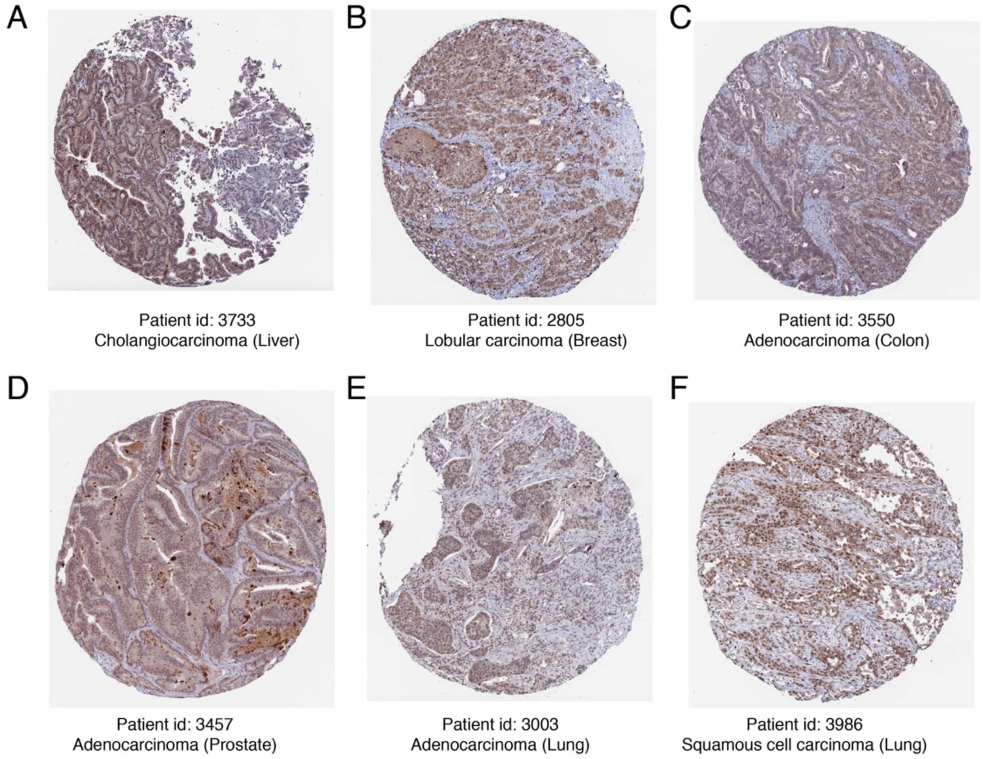

Supplementary Figure 6. Example of cancer tissues stained positive for TRA16 in HPA.

The protein expression of TRA16 was profiled by the Human Protein Atlas. Example positive staining was shown for liver cancer (A), breast cancer (B), colon cancer (C), prostate cancer (D) and lung cancer (E, F).
